# Silicon-Integrated Acid-Etched SnO_2_/N-CNT Composite as a High-Capacity Anode for Lithium-Ion Batteries

**DOI:** 10.3390/nano16100622

**Published:** 2026-05-18

**Authors:** Soghra Hosseini, Arunakumari Nulu, Keun Yong Sohn

**Affiliations:** Department of Nanoscience and Engineering, Center for Nano Manufacturing, Inje University, 197 Inje-ro, Gimhae 50834, Gyeongsangnam-do, Republic of Korea; sadathosseinisora1989@gmail.com (S.H.); venuaruna2@gmail.com (A.N.)

**Keywords:** SnO_2_ anode, silicon-incorporated acid-etched SnO_2_, nitrogen-doped CNTs, lithium-ion batteries, high-capacity anode

## Abstract

Herein, we report the rational design of an A-SnO_2_/Si@N-CNT nanocomposite, fabricated via facile ball milling followed by high-temperature annealing. In this design, surface-modified SnO_2_ (A-SnO_2_) serves as the primary active framework, silicon nanoparticles are introduced to enhance overall capacity, and nitrogen-doped carbon nanotubes (N-CNTs) provide a conductive and mechanically resilient network. The incorporation of silicon nanoparticles and N-CNTs into A-SnO_2_ facilitated the formation of strong Si–C and Si–O–Sn bonds, thereby improving electrical conductivity and structural stability and reinforcing interfacial interactions between the active materials and the conductive CNT matrix, resulting in superior electrochemical performance. Morphological analysis confirmed that the composite maintained structural stability without severe cracking after 100 cycles at 100 mAh g^−1^. The electrode delivered reversible capacities of 1002 and 622 mAh g^−1^ at 0.1 and 0.5 A g^−1^, with capacity retentions of 78.7% and 73.17%, respectively. Even at 1.0 A g^−1^, a stable capacity of 441 mAh g^−1^ with 80.96% retention was achieved. These findings demonstrate the effectiveness of coupling surface-modified SnO_2_ with Si- and N-doped carbon frameworks for advanced lithium-ion battery anodes.

## 1. Introduction

The growth of power tools, such as portable and electric devices, has made fast-charging batteries a worldwide research issue. Consequently, lithium-ion batteries (LIBs) have become crucial for energy storage over the past few decades due to their widespread use in portable electronics and their role in power systems [1,2,3]. Meanwhile, due to the limited capacity (~372 mAh g^−1^) of commercial graphite, researchers have conducted extensive investigations on anode materials, which are used in lithium-ion batteries [4,5,6]. The capacity limitation of current anodes and the increasing demand to improve next-generation storage systems have led us to develop new anode materials with higher capacity. Meanwhile, one of the attractive materials that has gained the attention of researchers in LIB applications is tin(IV) oxide (SnO_2_). Its high theoretical capacity of ~1494 mAh g^−1^, cost-effectiveness, non-toxicity, and low operating voltage plateau are the main reasons [7,8,9]. Although this material has its own advantages, it faces serious disadvantages. The practical application of SnO_2_ remains hindered because wide-bandgap semiconductors have intrinsically low electrical conductivity, which impedes charge transfer and leads to slow reaction kinetics, which is the nature of SnO_2_ [6,7]. Also, as with other high-capacity materials, SnO_2_ faces a notable volume expansion exceeding ~300%, which includes the pulverization of particles, mechanical fractures, and rapid capacity loss in lithium-ion batteries [7,10]. Extensive research on hybrid materials has addressed the inherent limitations of SnO_2_-based anodes, aiming to enhance their structural stability and electrochemical performance. Effective strategies that researchers have mostly focused on are, first, controlling the microstructure of SnO_2_ at the nanoscale [11] and, second, integrating nanostructured SnO_2_ with conductive materials such as carbon, metals, or polymers [3]. The advantages of nanoscale SnO_2_ include reducing tin aggregation, improving lithium storage capacity, shortening lithium-ion diffusion pathways, and providing a high surface area [3,12]. One effective way to enhance the structural and electrochemical performance of SnO_2_ nanoparticles is to introduce an acid during synthesis to control particle size [13] or after synthesis to modify the surface [12]. Sun et al. showed that the acid concentration, especially that of HCl, controls the size, structure, and morphology of hierarchical SnO_2_ nanostructures, leading to better performance. Adjusting acidity during synthesis enables precise control over nanostructural features [13]. And our prior work reported that post-synthesis acid treatment reduces nanoparticle size and creates defect-rich surfaces, yielding smaller particles with better dispersion. These features enable faster redox reactions, reduce polarization, and improve electrochemical performance, thereby enhancing lithium-ion storage behavior [12]. Moreover, integration with conductive carbon materials boosts electrical conductivity and stability. These matrices buffer volume changes, improve electron transport, enhance lithium storage, and improve electrical conductivity and electrochemical performance in tin(IV) oxide anodes [3,6,14]. In particular, carbon nanotubes offer distinct advantages, including high electrical conductivity, mechanical robustness, and a large specific surface area [12,15,16,17]. Furthermore, doping carbon with heteroatoms, such as nitrogen, introduces active sites for lithium-ion adsorption and diffusion [12,17,18], thereby increasing conductivity and capacity and improving the chemical and physical properties of materials [19,20,21]. Wang et al. synthesized amorphous carbon-coated Sn–SnO_2_ nanoparticles (~3 nm thick; 20–50 nm size) embedded in helical carbon nanotubes (~30 nm diameter) on Cu foam via the hydrothermal and CVD method. The C@Sn–SnO_2_/CNT electrode achieved a first-cycle capacity of 1772 mAh g^−1^ at 0.2 A g^−1^, maintaining 856 mAh g^−1^ after 300 cycles and delivering 480 mAh g^−1^ at 1 A g^−1^ [22]. Another method that effectively enhances SnO_2_-based anodes is adding or doping elements such as Ni, Fe, P, and Si to modulate the physical and chemical properties of the materials. Research has shown that the structural stability and electrical conductivity of SnO_2_-based anode materials can be improved by incorporating or doping specific elements [6,19]. Recent studies have identified, among the elements for doping and incorporation, silicon (Si) as a promising strategy for improving the electrochemical performance of SnO_2_-based anode materials [6,23]. Research indicates that Si improves the structural stability of SnO_2_-based anodes over multiple lithiation and delithiation cycles by creating strong chemical interactions within the SnO_2_ matrix. Furthermore, owing to its advantageous Li^+^ conductivity, Si is employed as a surface coating material [24,25]. Additionally, Si incorporation effectively mitigates the aggregation of SnO_2_ and Sn nanoparticles, thereby further improving cycle stability [24,26]. For example, Shuai Liu et al. have reported enhancing the cyclic stability and fast charging of a sandwich-structured C/Si@SnO_2_ through ball milling, which incorporates a porous silicon (Si) buffer layer placed between the carbon layer and the inner layer of SnO_2_ through the use of ball milling. They produced N- and P-co-doped carbon with a disordered structure by directly carbonizing a mixture of ionic liquid and sawdust. The C/Si@SnO_2_ composite demonstrated excellent electrochemical performance when utilized as an anode, with a notable capacity of 919.21 mAh g^−1^ at a current density of 0.1 A g^−1^. Also, ball milling is cost-effective and eco-friendly for synthesizing, which uses mechanical energy to reduce particle size, mix phases, and enable solvent-free synthesis. It also increases the order and disorder area, aiding lithium-ion diffusion [27]. Furthermore, Xianqing Liang et al. [6] successfully fabricated a novel nanocomposite comprising silicon-incorporated SnO_2_ with graphene sheets (STOG). Through a simple hydrolysis process, ultrafine Si-incorporated SnO_2_ (STO) nanoparticles were uniformly distributed onto graphene sheets. It has been observed that Si incorporation enhances the Si-O-Sn bonding within the SnO_2_ matrix and strengthens the C-O-Sn bonding between STO and graphene, resulting in improved structural stability and electron/ion transport properties of the STOG nanocomposite, facilitating the reversible conversion of Sn–SnO_2_.

Taking into account the above-mentioned challenges and key factors, herein we synthesized a Si-incorporated acid-etched SnO_2_ and N-CNT composite (A-SnO_2_/Si@N-CNT) by a simple technique involving acid etching followed by ball milling and post-heat treatment, in which acid-etched SnO_2_ NPs and Si NPs were successfully integrated into an N-CNT matrix. As expected, the prepared composite anode showed excellent electrochemical properties and rate capability. Moreover, it showed better Li diffusion properties. Even at higher current densities, it performed well.

## 2. Experimental Section

### 2.1. Materials and Methods

Tin(IV) chloride pentahydrate (Sigma-Aldrich ≥ 98%, St. Louis, MI, USA), silicon nanocrystalline powder (Alfa Aesar, Ward Hill, MA, USA, ≤50 nm), melamine (Daejung, Siheung-si, Republic of Korea, ≥99%), MWCNTs (Hanwha Nanotech Corp., Seoul, Republic of Korea, CM-100, diameter: 10 nm–14 nm), hydrofluoric acid (Sigma-Aldrich), ethanol (Sigma-Aldrich, ≥99.5%), hydrochloric acid (Duksan pure chemicals, Ansan, Republic of Korea), and ammonia (NH_3_) (Daejung chemicals and metals Co., LTD, Siheung-si, Republic of Korea) were purchased and used without any further treatment.

### 2.2. Synthesis of A-SnO_2_/Si@N-CNT Composite

Acid-etched SnO_2_ nanoparticles (A-SnO_2_) and N-doped CNTs were prepared as reported in our earlier work [12,28]. A-SnO_2_ nanoparticles were prepared using a two-step procedure involving chemical co-precipitation [29], followed by surface acid etching, where SnCl_4_·5H_2_O (tin(IV) chloride pentahydrate) was used as the tin precursor, with HCl (hydrochloric acid) and ammonia (NH_3_) as solvents. After drying and thermal annealing, the obtained SnO_2_ nanoparticles were subjected to a controlled hydrofluoric acid (HF) treatment to induce surface etching. This post-treatment modified the surfaces of the SnO_2_ nanoparticles, leading to reduced crystallite size and altered surface morphology while maintaining the dominant SnO_2_ crystalline phase. The resulting acid-etched sample was denoted as A-SnO_2_. Furthermore, N-doped multi-walled carbon nanotubes (N-CNTs) were synthesized following the method reported by Nulu et al. [30], using melamine as the nitrogen source. In this process, pristine MWCNTs and melamine were mixed at a mass ratio of 1:3, ball-milled, and subsequently annealed at a high temperature under a nitrogen atmosphere. The obtained N-CNTs were collected after cooling and employed as the conductive carbon matrix. To synthesize the A-SnO_2_/Si@N-CNT composite, initially, the as-prepared A-SnO_2_ NPs and silicon NPs were mixed in a 2:3 ratio and ball-milled in a mini ball mill for 5 min to ensure thorough mixing. This mixture was named A-SnO_2_/Si NPs. Next, nitrogen-doped carbon nanotubes (N-CNTs) synthesized earlier were added to the A-SnO_2_/Si NPs mixture in a 1:1 ratio. The composite was then ball-milled for 8 h in a mini ball mill operating at an oscillation speed of 42 Hz. The obtained composite was placed in an alumina crucible and subjected to thermal treatment, following the previously reported conditions (600 °C, 2 h, an argon atmosphere, and a heating rate of 5 °C min^−1^) [12]. After heating, the tube furnace was cooled to room temperature, and the final composite was collected and labeled as A-SnO_2_/Si@N-CNT. To know the effect of the N-CNTs, the electrochemical properties of A-SnO_2_/Si were also studied and compared with those of A-SnO_2_/Si@N-CNT.

### 2.3. Material Characterization

The structural properties of the synthesized materials were characterized by XRD spectroscopy with a Cu Kα radiation source (λ = 1.54 Å) (10° ≤ 2θ ≤ 80°), employing a Rigaku D/MAX-2200 Ultima (Rigaku Corporation, Tokyo, Japan). Thermogravimetric analysis (TGA) (Shimadzu, Kyoto, Japan (DSC-60/DTG-60) was performed in an air atmosphere at a heating rate of 10 °C min^−1^ and temperature range of 25 °C_800 °C. X-ray photoelectron spectroscopy (XPS) (AXIS SUPRA+/1) (United Kingdom (UK)) was performed to analyze the element states. Raman spectroscopy was performed (JP/NRS-3300/wavenumber (~57 cm^−1^) (Japan)). Surface morphology was examined via scanning electron microscopy (SEM; Merlin compact/0.8 nm @ 15 kV, 1.6 nm @ 1 kV, London, UK) and high-resolution transmission electron microscopy (HR-TEM; JEOL, Akishima, Japan (JEM-F200)) equipped with an energy-dispersive X-ray spectroscopy (EDX) detector.

### 2.4. Electrochemical Measurements

The working electrode was prepared by uniformly mixing the as-synthesized active material, Super-P (conductive additive), and a polyamideimide (PAI) binder dissolved in N-methylpyrrolidone (NMP) in a mass ratio of 70:15:15%. The mixture was milled with a mini ball mill for 30 min at an operating oscillation speed of 40 Hz. The resulting slurry was uniformly coated onto a copper foil substrate by a doctor blade to a controlled thickness of 25 mm (~25 µm), and then, in order to produce the final electrode, it was dried in two steps: first, to remove NMP, it was heated at 90 °C in a normal oven, then placed in a vacuum oven, and heat treatment was performed at 200 °C for 3 h. Electrochemical characterization was conducted using a half-cell (CR2032) with a mass loading of ~2.03 mg cm^−2^. The electrodes were assembled within an argon-filled glove box. Lithium foil served as the counter electrode, a polypropylene membrane functioned as the separator, and the electrolyte was composed of 1 M LiPF6 in a 1:1 *v*/*v* mixture of ethylene carbonate (EC) and diethyl carbonate (DEC). Galvanostatic charge–discharge tests were conducted within a voltage window of 0.01 to 3.00 V. Cycling stability was determined at a current density of 0.1 A g^−1^, and the rate capability was evaluated across 0.1 to 1.6 A g^−1^. Cyclic voltammetry (CV) and electrochemical impedance spectroscopy (EIS) measurements were carried out on a BioLogic SP-150-127 electrochemical workstation at potentials from 0.01 to 3.0 V. Impedance spectra were acquired over a frequency range of 10 mHz to 10 kHz. A BioLogic battery testing instrument at room temperature was used to measure all of the electrochemical properties.

## 3. Results and Discussion

### 3.1. Experimental Synthesis Mechanism

**Figure 1:**
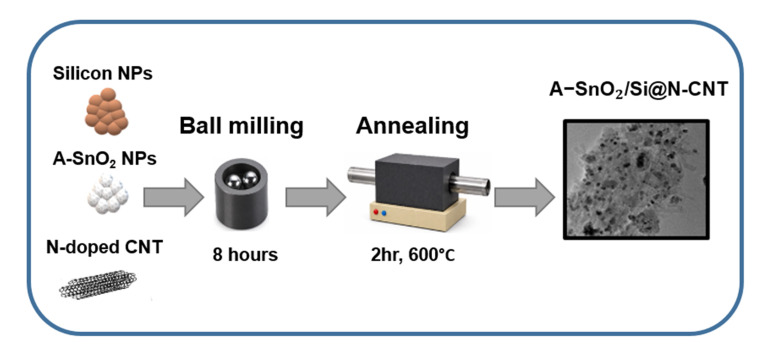
Stepwise synthesis process of A-SnO_2_/Si@N-CNT composite.

The schematic of the preparation of the A-SnO_2_/Si@N-CNT composite is shown in Figure 1. First, silicon nanoparticles were combined with an appropriate quantity of A-SnO_2_ NPs and subjected to ball milling for 5 min at a rotational speed of 42 Hz. During this process, the A-SnO_2_ and Si NPs were dispersed thoroughly, forming a uniform mixture. To facilitate the attachment of A-SnO_2_ and Si crystalline nanoparticles onto the N- doped CNTs, the A-SnO_2_/Si mixture was then distributed within N-doped CNTs and ball-milled for hours. Then, following the previously reported annealing conditions [12] (600 °C for 2 h at a heating rate of 5 °C min^−1^), the mixture underwent heat treatment to promote bonding among the A-SnO_2_, Si, and N-CNTs. The nanoparticles are uniformly dispersed and strongly coupled within the CNT framework, enabling efficient electron transport and structural stability. The N-CNT-to-SnO_2_/Si mass ratio was set to 1:1 to balance electrical conductivity and structural stability against the increased volume expansion introduced by Si, while avoiding excessive dilution of the electrochemically active components [31].

### 3.2. Structure, Morphology and Component Analysis

**Figure 2:**
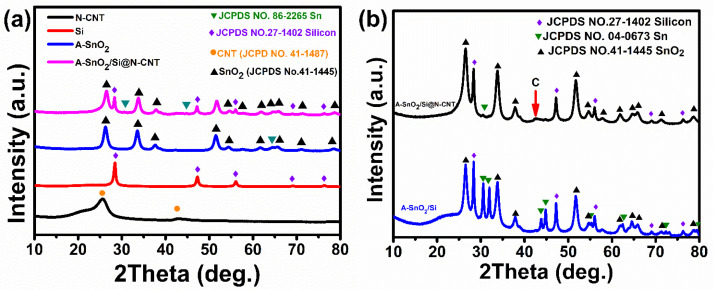
(**a**) X-ray diffraction patterns of N-CNT, A-SnO_2_ Reproduced with permission from Ref. [12]Copyright 2026 Elsevier, Si and A-SnO_2_/Si@N-CNT; (**b**) X-ray diffraction patterns of A-SnO_2_/Si and A-SnO_2_/Si@N-CNT nanocomposites.

The XRD patterns of the prepared A-SnO_2_/Si and A-SnO_2_/Si@N-CNT materials are shown in Figure 2a,b. The XRD patterns of acid-etched SnO_2_ (A-SnO_2_) and N-doped CNTs, which have been previously reported in our earlier work and are replotted here for comparison purposes, are shown in Figure 2a [12,30]. As discussed in the previous study, the diffraction peaks of the as-prepared A-SnO_2_ show characteristic diffraction peaks that match the peaks reported in the research for the following synthesis process [12,28,32]. Additionally, Figure 2a shows the diffraction peaks of the N-doped CNTs, with peaks at 25.6° and 43.39° for the (002) and (100) planes, reflecting the hexagonal graphite structure (JCPDS No. 41-1487) [12,33]. The X-ray diffraction pattern analysis of the A-SnO_2_/Si and A-SnO_2_/Si@N-CNT electrodes is presented in Figure 2b. The presence of SnO_2_ nanoparticles was confirmed by the formation of tetragonal SnO_2_, in line with the standard JCPDS No. 41–1445 and JCPDS card No. 88-0287. The diffraction peaks at 2θ values of 26.6°, 33.9°, 37.9°, 51.8°, 54.7°, 61.9°, 71.3°, and 78.7° correspond to the (110), (101), (200), (211), (220), (310), (202), and (321) crystal planes, respectively [,^12^,^28^,^29^]. For the A-SnO_2_/Si and A-SnO_2_/Si@N-CNT samples, the diffraction peaks at 2θ of 28.4°, 47.3°, 56.1°, 56.6°, 68.9°, and 76.3° are assigned to the (111), (220), (311), (400), (331), and (422) planes of Si (JCPDS No. 27-1402) [34]]. Nevertheless, the XRD patterns of both composites exhibited new diffraction peaks indexed to Sn (JCPDS No. 04-0673) and Sn (JCPDS No.86-2265), following ball milling. As previously reported, this transition from partial SnO_2_ to metallic Sn demonstrates the influence of mechanical energy on the phase composition of the materials during ball milling [27,35]. Using the reference intensity ratio (RIR) method, the inorganic phases in the composite were quantified, with Sn (~2.47 wt%), SnO_2_ (~34.36 wt%), and Si (~17.97 wt%), and the remaining ~45.2 wt% was attributed to carbon. According to the rule of mixtures, Si and SnO_2_ dominated the capacity contribution (~54.4% and ~43.4%, respectively), whereas Sn contributed only marginally (~2.1%) [36].

Furthermore, compared with A-SnO_2_/Si@N-CNT, the A-SnO_2_/Si electrode showed a broad peak between 21.6 and 28.4° in the 2θ diffraction pattern, which could be attributed to SiO_x_ formation [27]. This peak was not prominent in the A-SnO_2_/Si@N-CNT composite.

This could be a result of its overlap with the peak of N-CNTs at 25.6°, corresponding to JCPDS No. 41-1487, and the high-temperature annealing process [34]. Furthermore, a small bump at ~42.6° (red arrow) showed the carbon present in the A-SnO_2_/Si@N-CNT electrode [12].

**Figure 3:**
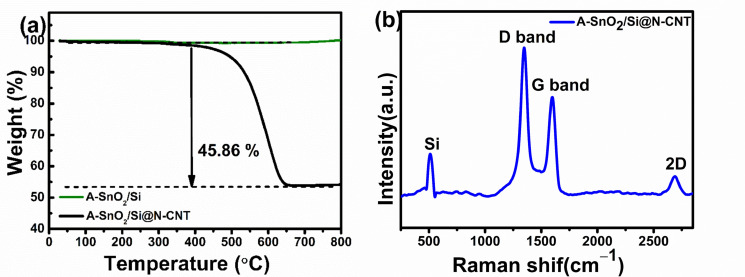
(**a**) Thermogravimetric analysis for A-SnO_2_/Si and A-SnO_2_/Si@N-CNT, and (**b**) Raman spectrum for A-SnO_2_/Si@N-CNT.

The TGA results for the weight loss profiles are shown in Figure 3a. First, no notable change was observed in the A-SnO_2_/Si composite. However, for A-SnO_2_/Si@N-CNT, pronounced weight loss was observed due to the combustion of carbonaceous species. The A-SnO_2_/Si@N-CNT composite exhibited a reducing mass content that began at around ~400 °C and ended at ~650 °C as a result of the decomposition of CNT carbon. As shown, because the process was carried out without a solvent, no notable weight loss below 150 °C was observed [12]. In the A-SnO_2_/Si@N-CNT composites, about 45.86% of the weight was lost at 600 °C. From 600 °C to 800 °C, the weight remained stable, and no additional mass loss occurred.

The Raman spectrum of A-SnO_2_/Si@N-CNT in Figure 3b depicts a peak at approximately 510 cm^−1,^ which is a characteristic peak of Si in the sample, and two peaks at 1348 cm^−1^ and 1595 cm^−1^, corresponding to the D-band and G-band, respectively. The D-band indicated the presence of a structural defect, while the G-band was associated with graphitic carbon [37]. The I_D_/I_G_ ratio is widely used to assess graphitization in carbon materials and evaluate defects in N-doped carbon nanotubes (N-CNTs) [38]. The I_D_/I_G_ intensity ratio for A-SnO_2_/Si@N-CNT was calculated to be 1.59. The ball-milling process, the N-doping of the carbon skeleton, and the presence of Si during ball milling resulted in significant disorder in the carbonaceous materials, which could have improved the electrochemical performance and cycling durability [38]. The second-order D peak, which appeared as the result of double-resonant Raman scattering (two-phonon emissions), was located at ~2691 cm^−1^. As previously reported, this shows that the carbon skeleton (N-CNTs) becomes thinner than the N-CNTs before the ball-milling process [39].

**Figure 4:**
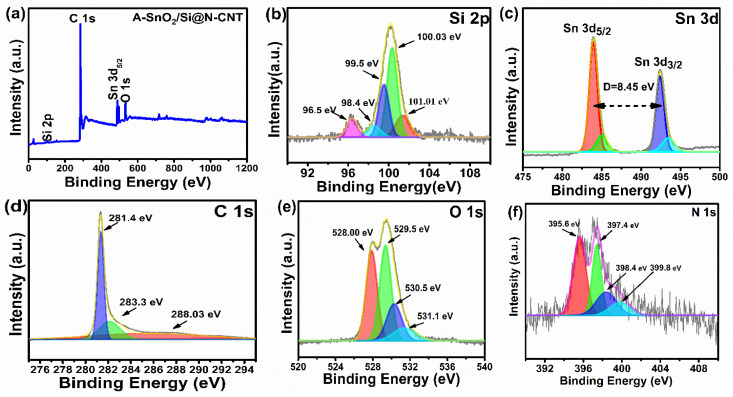
XPS spectra: (**a**) survey scan of A-SnO_2_/Si@NCNT; high-resolution XPS spectra: (**b**) Si 2p, (**c**) Sn 3d_5/2_, (**d**) C 1s, (**e**) O 1s, and (**f**) N 1s.

The XPS analysis is presented in Figure 4a–f to evaluate the chemical bonding in the A-SnO_2_/Si@N-CNT composite. The survey spectra depicted in Figure 4a confirm the presence of Si, Sn, O, C, and N materials. The high-resolution Si 2p spectrum (Figure 4b) shows two peaks at 98.4 and 99.5 eV, corresponding to the Si–Si bond [37,40]. The Si 2p peak at approximately 96.5 eV is interpreted as elemental silicon (Si^0^), with a negative chemical shift, resulting from a strong interaction with the N-doped CNT matrix, electron transfer from SnO_2_, and lattice distortion caused by ball milling. Similar Si 2p shifts (~97.1 to 97.7 eV) have been reported previously [41]. Furthermore, a peak at 100.3 eV was observed, which corresponded to Si-C bonding, and a peak for the Si-C bond was exhibited in the C1s spectra [42]. A peak at 101.01 eV is associated with SiO_x_ (0 < x ≤ 2), which could be attributed to Si–O–Sn, suggesting the good dispersion of Si NPs in the composite [6]. However, the much higher peak intensities of Si–Si and Si-C compared with silicon oxide suggest a low level of surface oxidation. Since the XRD results rule out silicon oxides, it can be concluded that only the surfaces of the silicon nanoparticles are coated with a thin, protective silicon oxide layer [43]. The high-resolution XPS spectrum of Sn 3d is shown in Figure 4c. The two intense peaks at 484 eV and 492.4 eV represent the Sn 3d_5/2_ and Sn 3d_3/2_ components, respectively. These peaks are separated by an 8.4 eV spin–orbit splitting, matching typical values for tetravalent tin (Sn^4+^), as previously reported in [44,45,46,47,48]. A detailed analysis of the Sn 3d _5/2_ region reveals that it splits into two components: one at 484 eV, corresponding to partially reduced or metallic tin (Sn^0^), and another at 485.1 eV, linked to Sn^2+^ species at 485.1 eV. Moreover, the Sn 3d _3/2_ peak also consists of two parts: one at 492.4 eV, which aligns with lower oxidation states of Sn (Sn^0^/Sn^2+^), and another at 493.5 eV, indicative of Sn^4+^ species [12,48]. The presence of multiple doublets shows that metallic tin (Sn^0^) exists alongside oxidized tin species (Sn^2+^ and Sn^4+^) within the composite. Such mixed oxidation states, including Sn^0^, Sn^2+^, and Sn^4+^, are frequently observed in tin-based composites and are crucial for improving their electrochemical performance. Sn^0^ enhances electronic conductivity, while the oxidized states (Sn^2+^ and Sn^4+^) facilitate lithium-ion storage through reversible conversion and alloying processes [12]. The C 1s spectra of the A-SnO_2_/Si@N-CNT composite (Figure 4d) can be separated into three peaks, located at 281.4 eV, 283.3 eV, and 288.03 eV, respectively, representing the metal–C (Si–C or Sn–C), C=N/C–N, and C=O–C bonds [12,39,49,50]. The robust (Si–C) bond between silicon and nitrogen-doped carbon nanotubes (N-CNTs) helps maintain the composite’s structural integrity during charge–discharge. Thus, the electrode delivers high capacity, excellent cycling stability, and high rate capability [12,42]. The high-resolution O 1s XPS spectrum of the composite (Figure 4e) is separated into four distinct peaks: 528.00 eV, Sn−O at 529.5 eV, Si-O/Sn-O at 530.5 eV, and Sn−O, C=O at 531.1 eV [12,37,48]. The presence of a Si-O bond suggests the doping of Si in the composite, which is present in the form of amorphous SiO_x_, which is very low, as observed in Si 2p spectra [51]. The peak at 528.5 eV likely indicates O^2−^ binding to Sn^4+^ in the SnO_2_ layer, suggesting partial surface oxidation of A-SnO_2_ nanoparticles. This passivation layer forms during acid treatment or ball milling, confirming SnO_2_ presence. These oxygen peaks indicate oxygen from both the SnO_2_ lattice and the surface functional groups of the carbon composite [45]. Additionally, the N 1s spectrum shows four peaks at 397.4, 398.4, and 399.8 eV, attributed to pyridinic N, pyrrolic N, and graphitic N, respectively [48,52]. A peak at 395.6 eV indicates the formation of Sn–N bonds, likely due to high-temperature annealing [12,45,53]. These findings confirm the nitrogen inside the composite. This introduction of N atoms into the carbon skeleton not only improves the storage capacity of CNTs but also enhances their conductivity. These factors can improve rate performance and stability during cycling [12,54]. Furthermore, the relative quantities of these four states from the deconvoluted N 1s spectrum were as follows: pyridinic N was dominant (27.28%), followed by pyrrolic N (19.7%), graphitic N (11.21%) and Sn-N bonds (41.75%). These species enhance electrochemical performance by improving electronic conductivity and providing additional active sites, thereby facilitating lithium-ion storage and kinetics [55].

**Figure 5:**
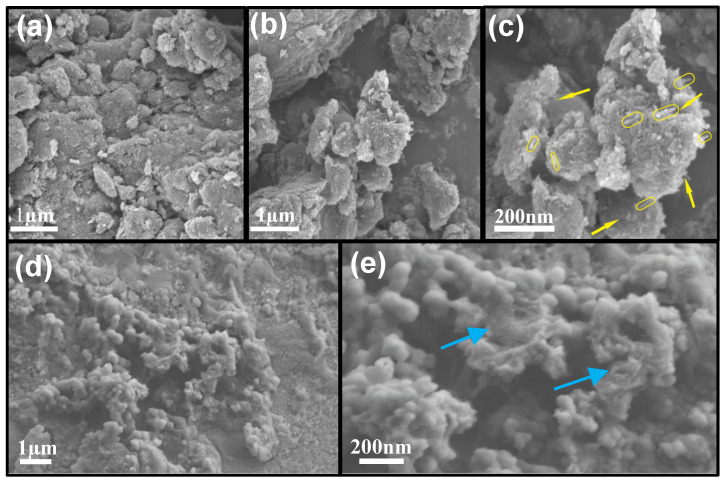
Scanning electron microscopy images of A-SnO_2_/Si@N-CNT (**a**–**c**) before and (**d**,**e**) after long-term cycles at 100 mAh g^−1^.

The microstructures of the A-SnO_2_/Si@N-CNT composite were also examined by SEM and TEM analysis. Figure 5a–e shows SEM images of the A-SnO_2_/Si@N-CNT composite at 1 μm and 200 nm before and after 100 cycles at 100 mAh g^−1^. The images before cycling (Figure 5a–c) highlight a strongly interconnected structure of A-SnO_2_, silicon NPs, and N-CNTs, resulting in a more uniform composite. As previously reported in other research [56], extended ball milling may cause the external layer of N-CNTs to be exfoliated into fewer-walled structures. Figure 5c shows N-CNTs of different sizes, marked by yellow arrowes and yellow ovals. The Si and A-SnO_2_ NPs show uniform dispersion and close contact with N-CNTs. High-resolution SEM confirmed the absence of clusters or agglomerates. This observation indicates that many different sizes of N-CNTs are wrapped around the Si and A-SnO_2_ materials. The SEM images after cycling (Figure 5d,e) showed that the composite retained its surface structure, with minimal extreme cracking, thereby maintaining electrode integrity and stability during repeated lithiation–delithiation. Figure 5e shows some areas even after 100 cycles, where small particles and N-CNTs are clearly visible and strongly connected to each other and are marked by blue arrows. Notably, despite sustained exposure to the electrolyte and repeated volume fluctuations during repeated cycling, the electrode maintains its structural integrity, demonstrating effective resistance to degradation and particle detachment. This observation is further supported by the SEM-EDS mapping, which shows a uniform elemental distribution and indicates the successful integration of the active components in the composite (Figure S1).

**Figure 6:**
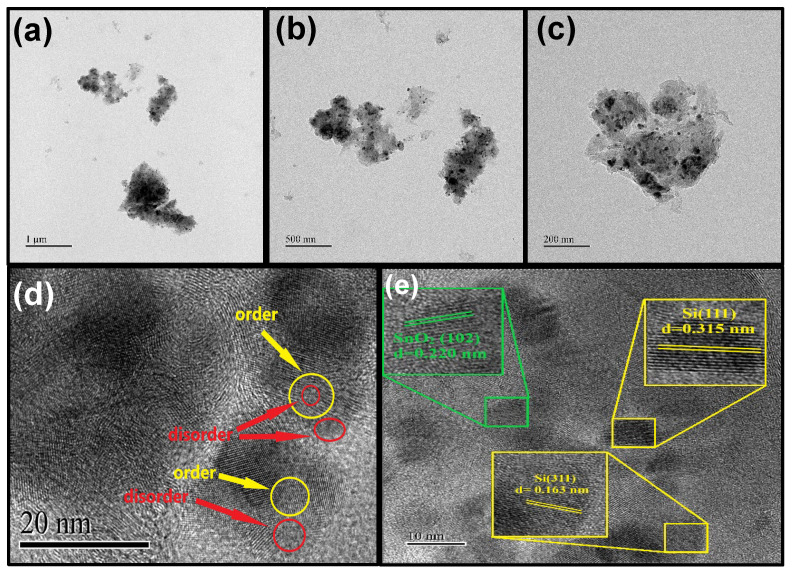
(**a**–**c**) TEM images of A-SnO_2_/Si@N-CNT at different magnifications, (**d**) HR-TEM image with disordered areas, and (**e**) lattice fringes of a selected area from the image.

Figure 6a–c shows typical low-magnification TEM images of A-SnO_2_ and Si nanoparticles dispersed and interconnected within the N-CNT network. As observed in the images, varying sizes of N-CNTs connect to each other and are then wrapped around other N-CNTs and surround the A-SnO_2_ and Si nanoparticles. This structure is observed in the SEM images, which show N-CNTs of varying sizes within the composite [56]. Ball milling of Si and A-SnO_2_ creates a mixed region with both of disorder (red circles) and order (yellow circles) structures (see Figure 6d) [27]. The HRTEM image in Figure 6e shows interplanar distances of about 0.315 nm and 0.163 nm, corresponding to the (111) and (311) crystallographic planes of silicon, and a lattice spacing of 0.220 nm related to the (102) plane of SnO_2_, indicating a well-defined crystal structure. Elemental analysis via EDS mapping, as shown in Figure S2a–f, confirms the uniform distribution of Si, Sn, C, O, and N across the material, demonstrating that the A-SnO_2_ nanoparticles are evenly anchored onto the N-CNTs.

## 4. Electrochemical Evaluation in LIBs

**Figure 7:**
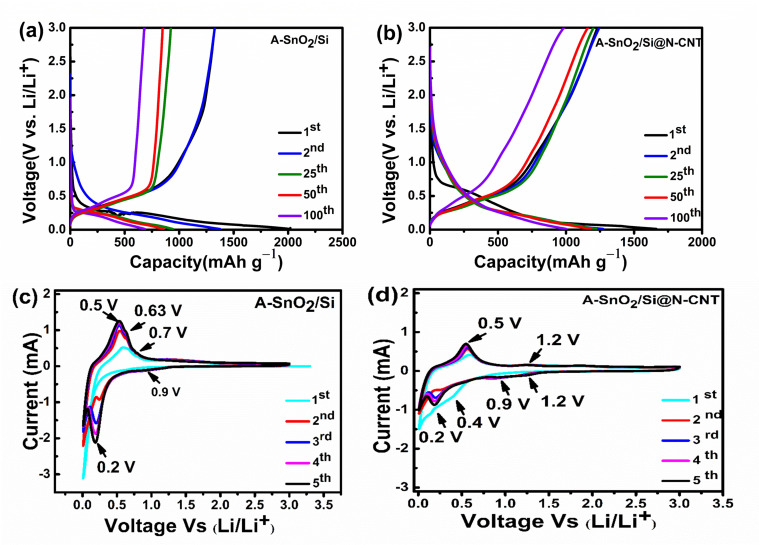
Charge/discharge profiles: (**a**) A-SnO_2_/Si and (**b**) A-SnO_2_/Si@N-CNT at 0.1 A g^−1^; CV curves from 0.01 V to 3.0 V: (**c**) A-SnO_2_/Si and (**d**) A-SnO_2_/Si@N-CNT.

The galvanostatic charge–discharge profile vs. voltage plots of the A-SnO_2_/Si and A-SnO_2_/Si@N-CNT composites for the first, second, 25th, 50th, and 100th cycles were evaluated at a current of 0.1 A g^−1^, as depicted in Figure 7a,b. The charge/discharge profile of the A-SnO_2_/Si material (Figure 7a) shows initial discharge and charge capacities of 2020 mAh g^−1^ and 1326 mAh g^−1^, respectively. In contrast, Figure 6b displays the corresponding profiles for A-SnO_2_/Si@N-CNT, revealing initial discharge and charge capacities of 1663 mA h g^−1^ and 1230 mA h g^−1^, respectively, resulting in initial coulombic efficiency (ICE) values of 65.64% and 73.94% and irreversible capacity losses (ICLs) of 694 mAh g^−1^ and 433 mAh g^−1^, respectively. Despite showing higher capacity in the first cycle, the A-SnO_2_/Si electrode experienced capacity fade sooner, which could be due to insufficient mechanical stability and electronic conductivity [6,34,57]. As observed in Figure 7a,b, for both the A-SnO_2_/Si and A-SnO_2_/Si@N-CNT composites, the initial discharge curve shows a plateau at around 0.6–0.89 V, corresponding to SEI formation, followed by a plateau at approximately 0.5 V, attributed to lithium alloying with Sn, and a long plateau near 0.08 V due to the alloying of Li^+^ with Si particles [27,58]. The formation of the SEI layer, with Li_2_O, was mainly responsible for the first notable irreversible capacity degradation [27,40,59]. After 50 cycles, A-SnO_2_/Si delivered capacities of 856/848 mAh g^−1^, with a capacity retention rate of 62.24%, and A-SnO_2_/Si@N-CNT maintained high reversible capacities of 1183/1160 mAh g^−1^, with a capacity retention of 92.9%. Notably, after 100 cycles, A-SnO_2_/Si displayed only 685/679 mAh g^−1^, with 49.8% capacity retention; meanwhile, the A-SnO_2_/Si@N-CNT electrode delivered 1002/983 mAh g^−1^, corresponding to a capacity retention rate of 78.73%. From the results, it was observed that the A-SnO_2_/Si@N-CNT composite showed an outstanding performance by adding N-CNTs, which prevented A-SnO_2_ nanoparticle agglomeration and effectively buffered the significant volume change [60]. As observed, the A-SnO_2_/Si@N-CNT composite demonstrated a more stable profile compared with the A-SnO_2_/Si composite in the 25th, 50th, and 100th cycles. From the results, it was observed that the A-SnO_2_/Si@N-CNT composite showed an outstanding performance by adding Si and N-CNTs, which prevented A-SnO_2_ nanoparticle agglomeration and effectively buffered the significant volume change [60]. The incorporation of Si increased the capacity, thereby improving both electrochemical reversibility and structural stability. It is worth mentioning that this specific one-dimensional structure of CNTs offers a shorter and direct pathway for ions and enhances the conductivity, which causes better storage capacity and durability [12]. Another role of N-CNTs is controlling the volume changes in SnO_2_ and silicon, maintaining structural stability, as shown by the poor cyclic performance stability of electrodes without N-CNTs. The findings suggest that in this composite, the lithium storage capacity of tin oxide (SnO_2_)-based anodes may be improved by combining silicon and nitrogen-doped carbon nanotubes (N-CNTs) [6,22,27,60,61]. In addition, the peaks of Si-C and Sn-C, with Si-O-Sn and Si-O-C bonds, effectively reduce volume fluctuations in silicon and SnO_2_, thereby contributing to the improved durability of the electrode.

Figure 7c,d illustrates the cyclic voltammetry (CV) profiles of the A-SnO_2_/Si and A-SnO_2_/Si@N-CNT electrodes, recorded over a voltage range of 0.01–3.00 V. As reported in previous research works, SnO_2_-based anodes exhibit a two-step lithiation mechanism in (LIBs) [62], consisting of an initial irreversible conversion to Sn and Li_2_O, followed by a reversible Li–Sn alloying/dealloying reaction, yielding Li_x_Sn (0 ≤ x ≤ 4.4). The equations below demonstrate that the electrochemical reactions of the A-SnO_2_/Si and A-SnO_2_/Si@N-CNT electrodes are as follows:

(1) $${SnO}_{2}+X{Li}^{+}+4e^{-}\to Sn+2{Li}_{2}O$$

(2) $$Sn+X{Li}^{+}+Xe^{-}↔Li_{X}Sn(0\leq X\leq 4.4)$$

(3) $$C\left(CNTs\right)+X{Li}^{+}+{Xe}^{-}↔{LiC}_{X}$$

(4) $$\left(XSi+{Li}^{+}+{Xe}^{-}↔{Li}_{X}Si\right)$$

In the A-SnO_2_/Si composite (Figure 7c), no reduction peak was observed in the first cycle. However, from the second to fifth cycles, two distinct peaks emerged at 0.9 V and 0.2 V. The peak at 0.2 V is attributed to the formation of Li-Sn alloy [40,63]. A weak peak at 0.9 V was observed, linked to the reduction of Sn oxides to metallic Sn and Li_2_O (Equation (1)), as noted in previous research [64]. Additionally, the oxidation peaks at approximately 0.5 V and 0.63 V are mainly assigned to the dealloying reaction of Li_x_Si, while the peak at 0.6 V relates to the dealloying reaction of Li_x_Sn, aligning with earlier studies [40,63,65]. In A-SnO_2_/Si@N-CNT in the cathodic scan (Figure 7d), the initial cycle shows weakly distinct peaks that are located at 0.7 V, 0.4 V, and 0.15 V (refs. [,^27^,^39^,^43^,^51^]), linked to the irreversible reduction of SnO_2_ to Sn, along with the formation of a solid-state electrolyte interface (SEI) film and the formation of Li_x_Sn alloy as a result of Li insertion into Sn, and the last one is attributed to Sn and Si alloy reaction with Li, respectively. However, the peak located at 0.7 V vanished in the following cycles as the SEI layer was formed on the surface of the active material [51]. From the second and fifth cycles, a broad peak was observed at around 1.0 V, which appeared to result from overlapping peaks at 0.88 and 1.2 V. The conversion of SnO_2_ to SnO/Li_2_O and SnO to Sn/Li_2_O can be associated with these peaks [12,51]. In contrast, the peak at 0.4 V shifted slightly to 0.2 V and became sharper, indicating the alloying reaction of Li_x_Sn [12,51]. From the second to the fifth cycles, a peak near 0.01 V was observed, which belonged to the formation of LiC_6_ due to lithium’s intercalation into CNTs, as described in Equation (3) [12,66]. Furthermore, in the anodic scan (delithiation process), a significant peak at 0.59 V in the first cycle linked the de-alloying reaction of the Li_x_Sn phase to the metal Sn phase. Notably, this peak coincided with and overlapped with the silicon peak, which occurred at around 0.5 V and is described by Equation (4). And a relatively faint peak was detected at 1.2 V, indicating that the oxidation process of tin (Sn) resulted in the formation of SnO_2_, which has a high reversible capacity [27,40,43,51,67].

The XPS characterization conducted after electrochemical cycling provides detailed insights into the electrode’s surface chemistry. Figure S3b shows two peaks at 98.6 and 99.4 eV, corresponding to the Si–Si bond [37,40]. Furthermore, a peak at 100.3 eV was observed, corresponding to Si-C bonding, and a Si–C peak was also observed in the C1s spectra [42]. Figure S3c shows the Sn 3d spectra after long-term cycling, with two main peaks at ~484.5 eV and ~492.8 eV, corresponding to Sn 3d_5_/_2_ and Sn 3d_3_/_2_, respectively, with a spin–orbit splitting of ~8.3 eV, indicating the presence of Sn^4+^ as SnO_2_. Deconvolution confirmed the presence of mixed Sn oxidation states (Sn^0^, Sn^2+^, and Sn^4+^), indicating partial reduction of SnO_2_. No shifting was observed. After long-term cycling, the C 1s (Figure S3d) peaks were observed to be higher and clearer. The increased intensity of the C–C and C=O groups in the C 1s signal supports the presence of organic species derived from electrolyte decomposition [68]. This indicates preservation of the carbon framework, with only minor surface oxidation, attributed to SEI formation [68]. The high-resolution O 1s XPS spectrum in Figure S3e of the composite can be distinguished into three main components at ~528.7, 529.4, and 530.9 eV, corresponding to Sn–O and Si–O/Sn–O bonding [12,37,48]. The dominant peak at ~530.9 eV is attributed to lattice oxygen in SnO_2_, indicating strong Sn–O bonding [69]. Notably, no peak is observed at ~531 eV. The N 1s spectrum retains pyridinic (~397.0 eV) and pyrrolic (~398.1 eV) nitrogen species, confirming that the fundamental nitrogen configuration remains largely intact [68]. These results collectively show that the composite structure experiences anticipated changes at the interface while still maintaining its active lithium-reactive sites.

**Figure 8:**
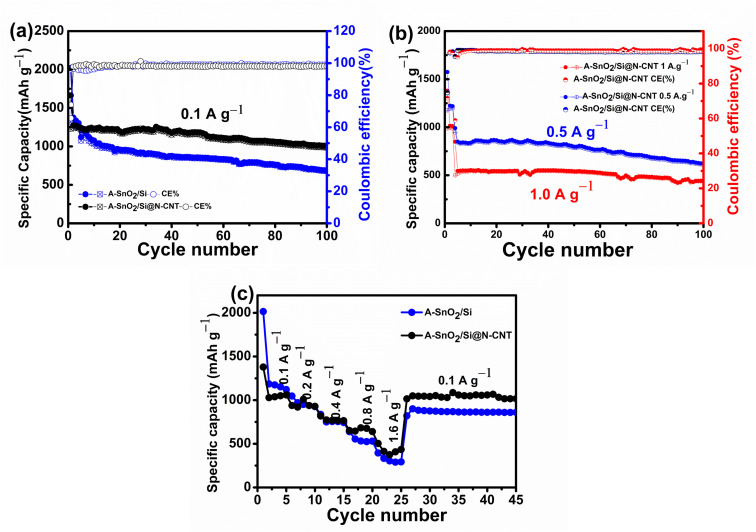
(**a**) Cycling performance and coulombic efficiency of A-SnO_2_/Si and A-SnO_2_/Si@N-CNT at 0.1 A g^−1^. (**b**) Cycling performance and coulombic efficiency of A-SnO_2_/Si@N-CNT composite at 0.5 A g^−1^ and 1.0 A g^−1^. (**c**) Rate performance at different current densities of 0.1 to 1.6 A $g^{-1}$.

Figure 8a compares the long-term cycling performance of the A-SnO_2_/Si and A-SnO_2_/Si@N-CNT electrodes. The A-SnO_2_/Si electrode starts with a high initial discharge capacity of 2020 mAh g^−1^ but experiences rapid capacity loss, dropping to 685 mAh g^−1^ after 100 cycles, which is a retention of only 49.8%. This capacity fade is primarily attributed to the direct contact with the electrolyte and significant volume expansion during lithium insertion/extraction, leading to structural fractures and particle pulverization. Conversely, the A-SnO_2_/Si@N-CNT electrode maintains better structural stability over 100 cycles. Therefore, N-CNTs act as a conductive carbon material for A-SnO_2_/Si, which not only reinforces the composite structure and mitigates large-scale fractures during lithiation/delithiation, but also enhances the conductivity of SnO_2_-based anodes. As a result, the A-SnO_2_/Si@N-CNT composite shows significantly improved cycling performance compared with A-SnO_2_/Si. After 100 cycles at 0.1 A g^−1^ within a voltage range of 0.01–3.00 V, it retains a high reversible discharge capacity of 1002/983 mAh g^−1^, with a notable coulombic efficiency. Furthermore, to investigate the ability of anode performance, long-cycle life studies with the resultant capacities of the A-SnO_2_/Si@N-CNT anode at high applied currents of 0.5 A g^−1^ and 1.0 A g^−1^ were carried out (see Figure 8b). To form a stable SEI layer, the first three cycles were performed at a lower applied current of 0.1 A g^−1^. The electrode exhibited initial specific discharge/charge capacities of 1574/1177 mAh g^−1^ and 1379/1015 mAh g^−1^, with ICEs of 74.78% and 71.64% (first specific capacity at 0.1 mAh g^−1^), respectively. After 100 cycles, the reversible capacities were 622/610 mAh g^−1^ with 73.17% and 441/436 mAh g^−1^ with 80.96% (capacity retention vs. second discharge capacity at 0.5 A g^−1^ and 1.0 A g^−1^, respectively). These findings verify the electrode’s structural stability and stable capacities. Figure 8c shows the rate performance tests of A-SnO_2_/Si and A-SnO_2_/Si@N-CNT at different current densities of 0.1, 0.2, 0.4, 0.8, and 1.6 A g^−1^. The A-SnO_2_/Si electrode composite demonstrated capacities of 2014,1047, 836, 637, and 394 mAh g^−1^ at current densities of 0.1, 0.2, 0.4, 0.8, and 1.6 A g^−1^, and a recovery of reversible capacity of 819 mAh g^−1^ when the current density was reduced from 1.6 A g^−1^ to 0.1 A g^−1^. Following high-rate cycling, A-SnO_2_/Si@N-CNT showed discharge capacities of 1378, 938, 817, 651, and 503 mAh g^−1^ at current densities of 0.1, 0.2, 0.4, 0.8, and 1.6 A g^−1^, respectively, and an impressive reversible capacity of 1015 mAh g^−1^, maintaining a retention of 97.82% after reverting to a current density of 0.1 A g^−1^. These results highlight the effectiveness of N-CNT incorporation in stabilizing the composite, demonstrating rapid Li-ion and electron diffusion, as well as outstanding reversibility and cycle stability. Furthermore, cross-sectional SEM analysis (Figure S4) showed that the electrode thickness increased from 22.9 to 54.24 μm after 100 cycles, indicating volume expansion. Despite this, the electrode maintained a sponge-like morphology, a continuous and porous structure without collapse or cycling-induced cracking, demonstrating effective stress buffering by the N-CNT network. EDS mapping (Figure S5) further verified the uniform elemental distribution after cycling. These results demonstrate that the N-CNT framework effectively accommodates volume expansion while maintaining its structural integrity and chemical stability during long-term cycling. Additionally, the introduction of N-doped MWCNTs improves the electron migration rate, provides extensive conductive pathways for rapid Li^+^ transport, and plays a significant role in controlling volume expansion [12,27,39]. The results also emphasize the synergistic contribution of Si NPs and N-CNTs within the composite. Si provides additional capacity and a more active reaction [51,59,70]. The results show that A-SnO_2_/Si@N-CNT exhibits excellent stability, making it a promising anode for future lithium-ion batteries.

The dQ/dV profiles in Figure S6a,b further illustrate the distinct electrochemical behaviors of A-SnO_2_/Si and A-SnO_2_/Si@N-CNT across the first, second, 50th, and 100th cycles. In the first cycle of A-SnO_2_/Si, two reduction peaks at 0.05 and 0.2 V are attributed to the formation of a Li−Si alloy, and the formation of a Li−Sn alloy also contributes to the peak at 0.2 V. During the first charge curve, two peaks at 0.2 and 0.5 V indicate the dealloying of Li_x_Si and Li_x_Sn, consistent with the previous report [27,65]. In contrast, for A-SnO_2_/Si@N-CNT, the same two reduction peaks at 0.08 and 0.2 V are observed. An additional peak at 0.6, related to SEI layer formation, disappears in subsequent cycles. The dealloying of Li_x_Sn is linked to ~0.4, and the oxidation of Sn to SnO is shown by the peak at 1.2 V. Compared with A-SnO_2_/Si, the A-SnO_2_/Si@N-CNT electrode exhibits more stable and well-defined dQ/dV peaks with significantly reduced polarization during cycling. The suppressed irreversible cathodic peak in the first cycle indicates mitigated SEI formation and improved interfacial stability. During prolonged cycling, N-CNTs may undergo minor surface reactions with electrolyte species, contributing to SEI formation. However, post-cycling XPS (Figure S3) shows that pyridinic and pyrrolic nitrogen species are largely preserved, with only slight surface oxidation observed in the C 1s spectrum, indicating good chemical stability [55]. Lithium storage is dominated by Li–Sn and Li–Si alloying reactions, while N-CNTs contribute only a minor capacitive component and primarily act as a conductive, mechanically robust framework [12]. In addition, post-cycling SEM (Figure S4) further confirms a continuous and interconnected structure without collapse, demonstrating effective buffering of volume changes. Overall, N-CNTs primarily serve as conductive supports and mechanical reinforcement, with limited contribution to charge storage, while maintaining structural and chemical integrity during long-term cycling. The highly overlapping lithiation/delithiation peaks over cycles demonstrate enhanced reaction reversibility and faster Li^+^ transport kinetics, which can be attributed to the conductive N-CNT network and effective buffering of volume changes [12]. Although the charge dQ/dV peak intensity of A-SnO_2_/Si@N-CNT slightly decreases after cycling, the highly overlapped peak positions and smooth profiles indicate stabilized delithiation kinetics and reduced polarization [27,65].

**Figure 9:**
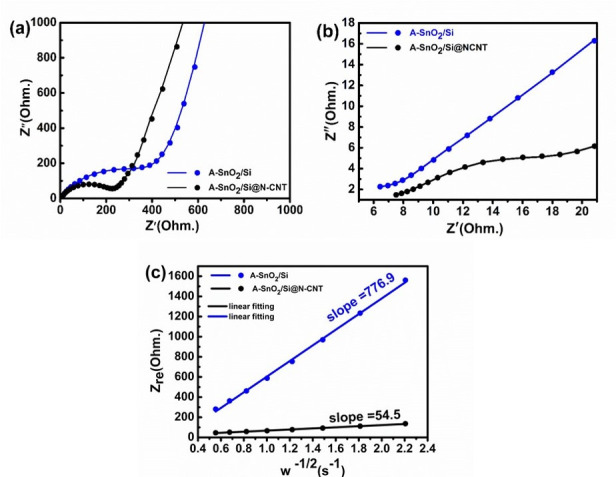
Nyquist plots of AC impedance for A-SnO_2_/Si and A-SnO_2_/Si@N-CNT electrodes (**a**) before and (**b**) after 10 cycles; (**c**) fitting results of the *Z_re_* vs. *w*^(−1/2)^ plots.

A comparative analysis of the electrochemical resistance of A-SnO_2_/Si and A-SnO_2_/Si@N-CNT, as derived from Nyquist plots obtained through AC impedance spectroscopy, is shown in Figure 9a,b. Each electrode curve shows depressed semicircles at middle and high frequencies and a slope at low frequencies, linked to solution resistance (R_s_), SEI resistance (R_SEI_), charge transfer resistance (R_ct_), and constant phase elements (CPE1 and CPE2). The low-frequency slope indicates Warburg impedance [30]. Figure S7 shows the equivalent circuit. Figure 9a,b shows the EIS measurements of the A-SnO_2_/Si and A-SnO_2_/Si@N-CNT composites, and the fitted values are listed in Table 1. As observed in Figure 9a, the A-SnO_2_/Si@N-CNT composites show a smaller diameter than that of the A-SnO_2_/Si electrode, which shows the rapid charge transfer process of the A-SnO_2_/Si@N-CNT electrode. The R_ct_ values drop markedly on the electrodes after 10 cycles, suggesting that the anode becomes activated, and new Sn particles form during phase transformation [30]. The lower R_ct_ of the A-SnO_2_/Si@N-CNT composite is due to the integration of Si, which enhances electron transfer and stability. Additionally, the carbon matrices facilitate Li-ion transport, provide charge-transfer pathways at the electrolyte–electrode interface, and create more active Li-ion pathways for charge/discharge [6,12,51,71,72]. XPS data also indicated that Sn/Si–carbon bonding could offer a rapid electron conduction pathway during charging and discharging [73]. To examine the sloped line associated with Li^+^ diffusion kinetics, Figure 9c shows the relationship between Z_re_ and ω^−1/2^. The fitted lines for *Z*′ versus ω^−1/2^ are used to analyze Li^+^ diffusion kinetics (Equation (5)). In the expression, *Z*′ represents the real part of the impedance, σ is the Warburg constant, and ω indicates the angular frequency [20,22]. The coefficient of lithium-ion diffusion is obtained by the Warburg constant (Equation (6)):

(5) $$Z^{\prime }=R_{s}+R_{ct}+\sigma \omega^{-1/2}$$

(6) $$D_{{Li}^{+}}=\frac{R^{2}T^{2}}{2A^{2}n^{4}F^{4}C^{4}\sigma^{4}}$$

Here, R denotes the gas constant, T is the absolute temperature, A is the electrode’s surface area, n is the number of electrons involved in the reaction, F is the Faraday constant, C is the lithium-ion concentration, and σ is the Warburg factor [74]. A smaller slope in the Warburg region suggests enhanced lithium-ion mobility and a higher diffusion coefficient *D^Li^*^+^. The calculated Li^+^ diffusion coefficient (D_Li+_) values, A-SnO_2_/Si, and A-SnO_2_/Si@N-CNT are presented in Table 1. The A-SnO_2_/Si@N-CNT composite exhibits a better value than the A-SnO_2_/Si electrode, showing the lower charge transfer resistance. According to the figure and values, the A-SnO_2_/Si@N-CNT electrode exhibits the lowest σ value, indicating a better Li^+^ diffusion behavior. This behavior may be attributed to the more disordered structures resulting from the addition of Si and N-CNTs [27]. The N-doped CNT framework functions as a support structure that effectively prevents the aggregation and pulverization of the A-SnO_2_ nanoparticles. It also aids in managing the stress and tension during lithiation and delithiation due to its excellent mechanical properties. Furthermore, the N-CNT framework offers more active sites and enhances electron transfer, leading to high lithium storage capacity. Moreover, the doped heteroatoms, such as N, help facilitate rapid lithium transport, improving the rate capability [12,22,61,72,73].

The Nyquist plots in Figure 9b demonstrate reduced semicircle features after cycling, indicating improved properties resulting from electrode activation [75]. Furthermore, a comparison between A-SnO_2_/Si@N-CNT and previous studies on SnO_2_-based anodes is provided in Table 2, which includes a comparison between A-SnO_2_ and A-SnO_2_@N-CNT. The A-SnO_2_/Si@N-CNT electrode shows markedly improved capacity and cycling stability, mainly due to additional lithium storage from Si alloying reactions. In contrast, A-SnO_2_@N-CNT exhibits only moderate improvement, arising from enhanced conductivity provided by the N-CNT network, which primarily acts as a conductive and structural support. These results highlight the key role of Si in enhancing electrochemical performance [12], where A-SnO_2_/Si@N-CNT demonstrated high capacity and outstanding stability, making it a promising durable anode material for LiB.

## 5. Conclusions

A silicon-incorporated, surface-modified SnO_2_ nanocomposite supported by nitrogen-doped carbon nanotubes (A-SnO_2_/Si@N-CNT) was successfully constructed via a co-precipitation strategy followed by ball milling and thermal annealing under an argon atmosphere. The rational integration of Si nanoparticles with acid-treated SnO_2_ and N-doped carbon nanotubes formed a highly interconnected composite architecture, in which robust interfacial bonds (Si–C, Sn–C, and Si–O–Sn) effectively reinforced structural integrity and mitigated mechanical degradation during repeated lithiation/delithiation cycles. Moreover, the N-CNT network established continuous electron pathways and significantly reduced Li^+^ diffusion resistance, thereby accelerating charge-transfer kinetics and improving electrochemical reversibility and long-term stability. However, incorporating N-CNTs slightly reduced the initial coulombic efficiency and specific capacity due to the limited SnO_2_/Si active materials. Benefiting from this synergistic structural and interfacial design, the A-SnO_2_/Si@N-CNT electrode delivered high reversible capacities of 1002 and 622 mAh g^−1^ at 0.1 and 0.5 A g^−1^, respectively, and maintained 441 mAh g^−1^ with 80.9% retention at 1.0 A g^−1^ after 100 cycles. These results demonstrate that the A-SnO_2_/Si@N-CNT nanocomposite is a promising anode material for next-generation, high-performance lithium-ion batteries, combining high capacity, excellent cycling durability, and scalable, eco-friendly synthesis.

## Figures and Tables

**Table 1:** Values of the equivalent circuit components data.

		Initial		After 10 Cycle			
Sample	R_S_ (Ω)	R_SEI_ (Ω)	R_ct_ (Ω)	R_S_ (Ω)	R_SEI_ (Ω)	R_ct_ (Ω)	D (${cm}^{2}s^{-1}$)
A-SnO_2_/Si	3.05	372	62.06	3.4	2.1	5.7	5.89 × 10^−14^
A-SnO_2_/Si@N-CNT	2.5	231	22.2	5.1	7.6	3.7	1.20 × 10^−11^

**Table 2:** Performance comparison between this work (A-SnO_2_/Si@N-CNT) and previous research about SnO_2_-based anodes.

Electrode Materials	No. of Cycles	Specific Discharge Capacity (mAh g^−1^)	Applied Current Density A g^−1^	Reference No.
SiySn1–yOx@C spheres	150	880.32	0.1	[23]
SiO_2_@SnO_2_/rGO	100	580	0.1	[26]
C/Si@SnO_2_	1000	919.21	0.1	[27]
Si@SnO_2_@C nanocomposite	300	554.3	0.5	[33]
Sheet-like SnO2@SiO_2_/graphite composite	80	1132	0.2	[39]
h-SnO_2_@Si-2	500	1030	0.1	[65]
Si/SnO_2_@CNFs-6	100	786.9	0.1	[67]
A-SnO_2_	100	425	0.1	[12]
A-SnO_2_@N-CNT	100	801	0.1	
This work	100	1002.6	0.1	
	100	622.04	0.5	
	100	441.14	1.0	

## Data Availability

The data presented in this study are available in the Supplementary Material.

## Supplementary Materials

The following supporting information can be downloaded at https://www.mdpi.com/article/10.3390/nano16100622/s1, Figure S1. Elemental mapping of A-SnO_2_/Si@N-CNT before cycling. Figure S2. (a–f) STEM images and corresponding EDX elemental mapping of A-SnO_2_/Si@N-CNT composite. Figure S3. XPS spectra: (a) survey scan of A-SnO_2_/Si@N-CNT; high-resolution XPS spectra: (b) Si 2p, (c) Sn 3d_5/2_, (d) C 1s, (e) O 1s, and (f) N 1s after long-term cycling. Figure S4. Cross-sectional SEM images for electrode changes (a) before and (b) after long-term cycling. Figure S5. Elemental mapping of A-SnO_2_/Si@N-CNT after long-term cycling. Figure S6. The differential charge capacity dQ/dV plots for (a) A-SnO_2_/Si and (b) A-SnO_2_/Si@N-CNT anodes. Figure S7. Equivalent circuit model of EIS fitting curve.
