# Supplementary material for: Silicon-Integrated Acid-Etched SnO2/N-CNT Composite as a High-Capacity Anode for Lithium-Ion Batteries
Source: Nanomaterials (Basel). 2026 May 18;16(10):622. doi: 10.3390/nano16100622 (PMC13209429; doi:10.3390/nano16100622)
Supplement: Supplementary file 1 [file nanomaterials-16-00622-s001.zip › nanomaterials-4293367-supplementary.pdf]

# Silicon-Integrated Acid-Etched $SnO_2$/N-CNT Composite as a High-Capacity Anode for Lithium-Ion Batteries

*Soghra Hosseini, Arunakumari Nulu, Keun Yong Sohn* *

Department of Nanoscience and Engineering, Center for Nano Manufacturing, Inje University, 197 Inje-ro, Gimhae, *Gyeongsangnam-do, 50834, Republic of Korea. E-mail:* ksohn@inje.ac.kr

## Supplementary Information

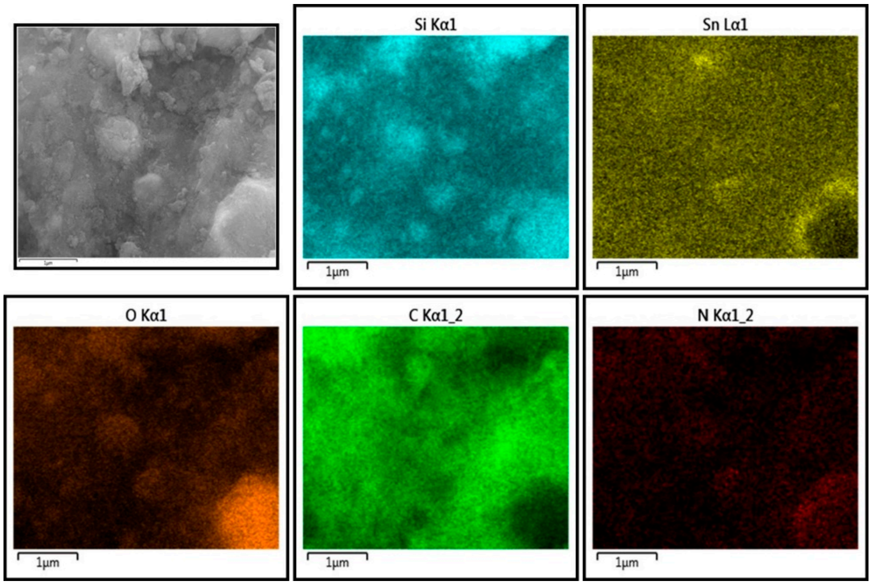


**Figure S1.** Elemental mapping of A-$SnO_2$/Si@N-CNT before cycling

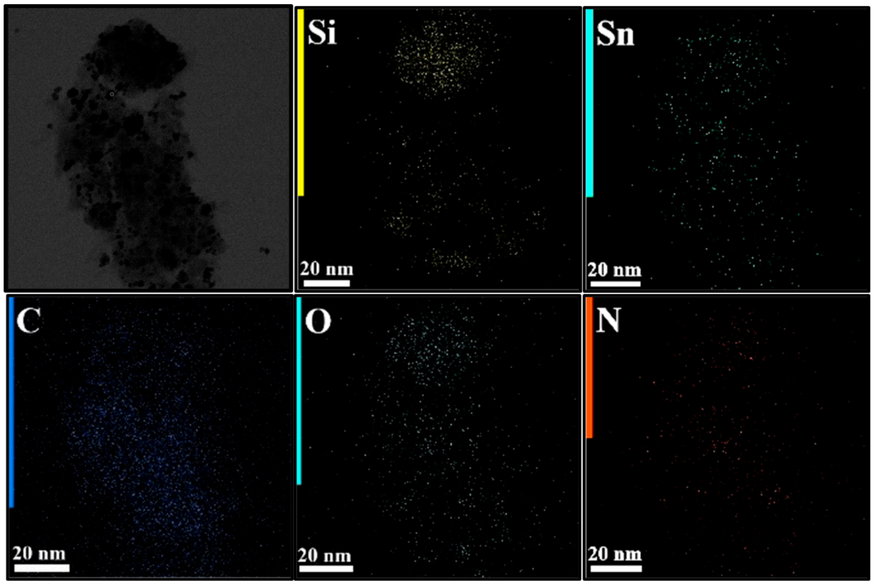


**Figure S2**. (a-f) STEM images and corresponding EDX elemental mapping of A-$SnO_2$/Si@N-CNT composite

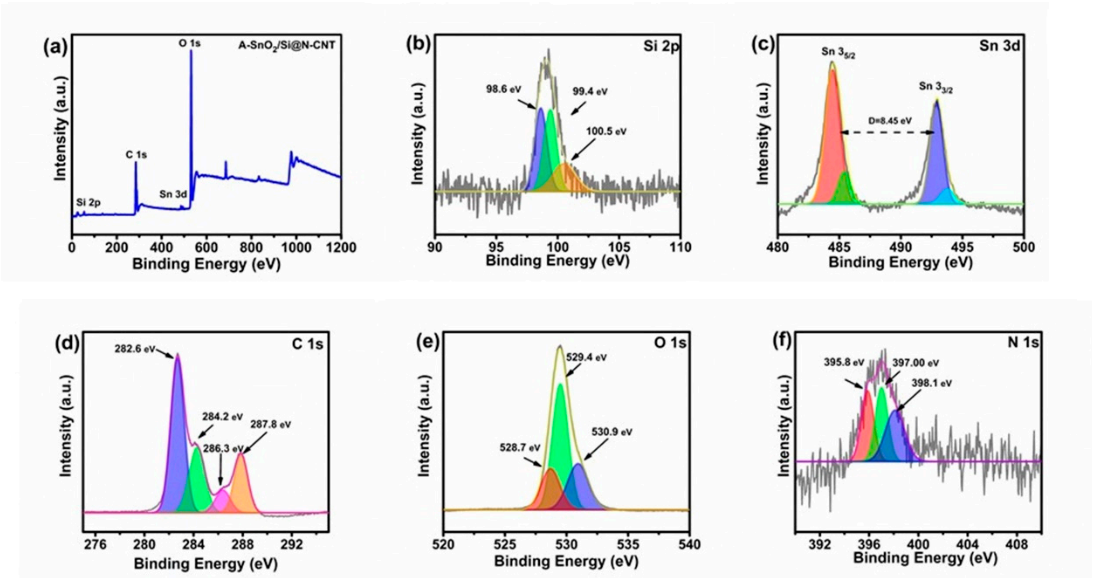


**Figure S3.** XPS spectra (a) survey scan of A-$SnO_2$/Si@NCNT; high-resolution XPS spectra (b) Si 2p, (c) Sn 3d5/2, (d) C 1s, (e) O 1s, (f) N 1s after long-term cycling

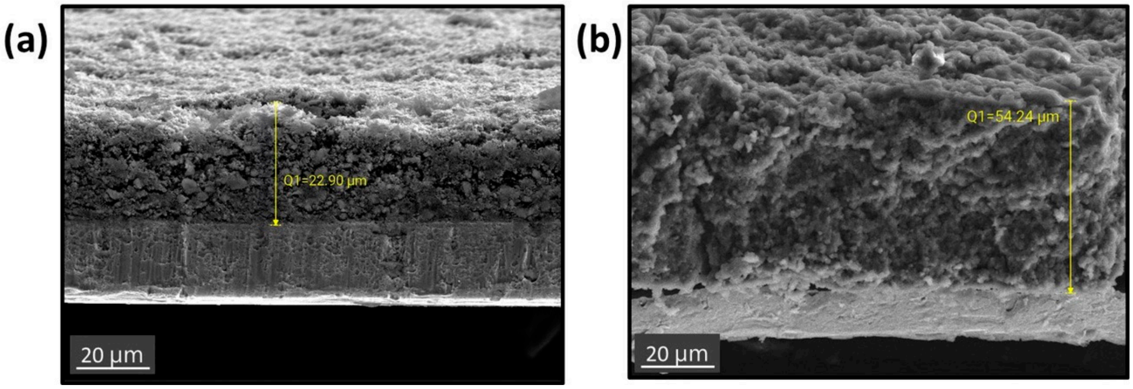


**Figure S4.** Cross-sectional SEM images for electrode changes (a) before and (b) after long-term cycling

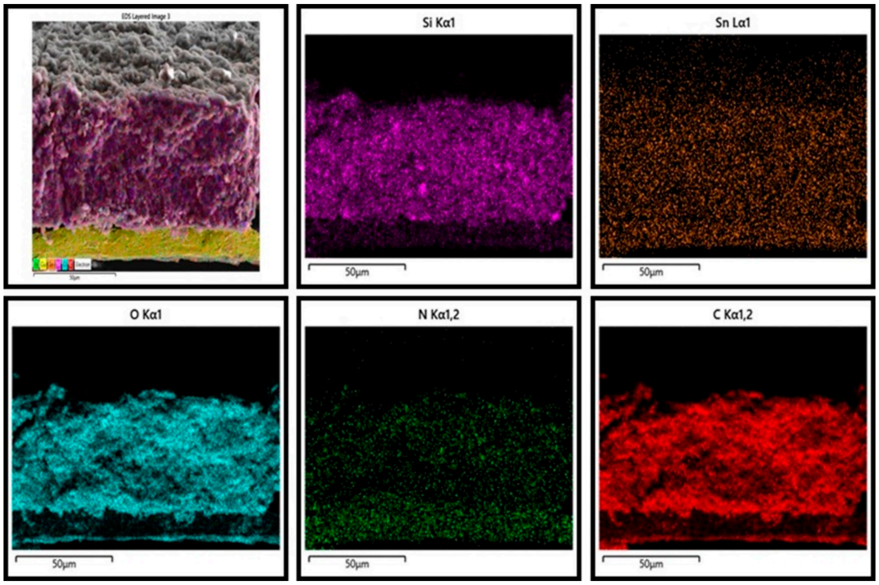


**Figure S5.** Elemental mapping of A-$SnO_2$/Si@N-CNT after long-term cycling

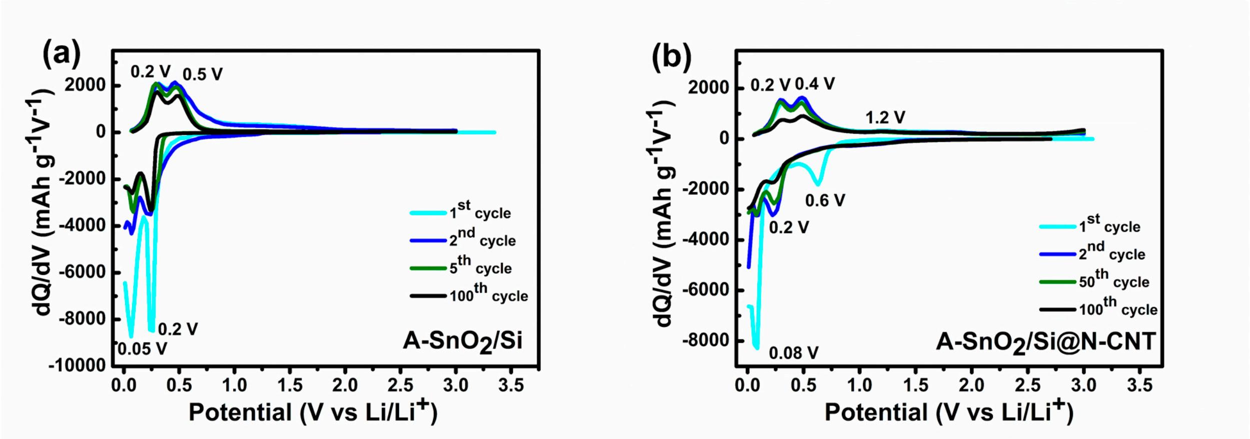


**Figure S6**.The differential charge capacity dQ/dV plots (a) A-$SnO_2$/Si and (b) of A-$SnO_2$/Si@N-CNT anodes

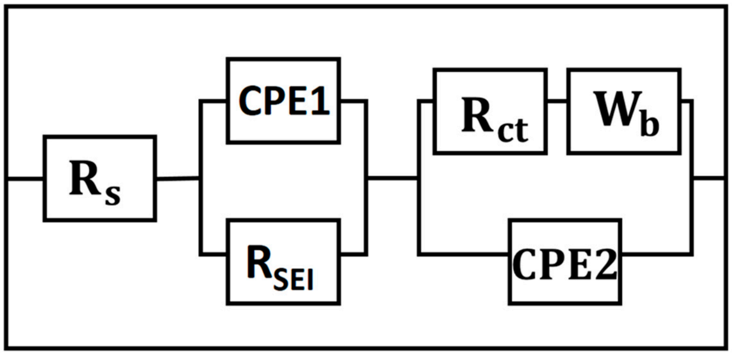


**Figure S7.** Equivalent circuit model of EIS fitting curve
